# Annual Commencement of the Baltimore College of Dental Surgery

**Published:** 1889-03

**Authors:** 


ANNUAL COMMENCEMENT OF THE BALTIMORE COLLEGE OF DENTAL SURGERY.—The forty-ninth annual commencement of the Baltimore College of Dental Surgery took place at the Lyceum Theatre, on Friday evening, March 15th, 1889, before a large audience. The exercises opened with an overture by Prof. Adam Itzel's orchestra, followed by prayer by the Rev. Wm. M. Dane. Prof. R. B. Winder, the dean, announced the names of the graduates and also conferred the degrees and prizes. The oration was delivered by J. N. McCormick and the valedictory by C. L. Morey.

The graduates were:

| | |
|---|---|
| G. E. Adams, | New Jersey |
| L. P. Brown, | New York |
| P. C. Carmichael, | New York |
| George B. Dorsey, | Mississippi |

| | |
|---|---|
| F. C. Exley, | Georgia |
| W. E. Hannah, | Pennsylvania |
| L. F. Hough, | Virginia |
| W. D. James, | Minnesota |
| A. L. Jones, | Virginia |
| N. B. Larkin, | Tennessee |
| J. E. Armitage, | New York |
| E. E. Butler, | Virginia |
| G. R. Carter. | Virginia |
| G. E. Coughlin, | Maryland |
| A. R. Eaton, | New Jersey |
| N. L. Hale, | Alabama |
| J. Hardy, | Virginia |
| H. L. Harlan, | Kentucky |
| G. W. S. Ireland, | Maryland |
| B. B. Johnston, | District of Columbia |
| W. C. Klatte, | South Carolina |
| A. Kraus, | Roumania |
| W. H. Lockwood, | Wisconsin |
| J. H. Minard, | Ontario |
| C. L. Morey, | Texas |
| F. H. Moore, | Maine |
| E. E. Murray, | North Carolina |
| J. F. McArthur; | Dakota |
| T. S. McElfish, | Maryland |
| O. M. Nisley, | Maryland |
| E. G. Powers, | Minnesota |
| J. P. Nesbitt, | Ohio |
| J. N. Penberthy, | Minnesota |
| J. G. Robinson, Jr. | Maryland |
| F. Rothenbach, | Germany |
| G. B. Rush, | Virginia |
| H. L. Sumption, | Virginia |
| J. R. Watson, | Pennsylvania |
| J. P. Whedbee, | Virginia |
| W. E. Walker, | Mississippi |
| W. R. Spencer, | Virginia |
| D. Williams, | North Carolina |
| C. Whitney, | District of Columbia |

The highest prize, a gold medal presented by the faculty, was given to J. P. Whedbee, of Virginia; honorable mention was made of W. H. Lockwood, Wisconsin, and F. Rothenbach, of Germany.

The first prize for mechanical dentistry, a set of forceps, from Snowden & Cowman, was awarded to George E. Coughlin, of Maryland, who also received as a first prize a dental engine, from the S. S. White Company, for operative dentistry. Honorable mention was made in operative dentistry of J. N. Penberthy, of Minnesota; W. E. Walker, of Mississippi; W. R. Spencer, of Virginia; Joseph Hardy, of Virginia, and F. C. Exley, of Georgia. The first prize for bridge-work, a set of Varney pluggers, from the S. S. White Company, was given to Wm. R. Spencer, of Virginia.

The alumni met in the afternoon at the college and elected the following officers: W. W. Walker, of New York, president; A. R. Eaton, vice-president; R. Baley Winder, Jr., secretary, and T. S. Waters, treasurer. The board of visitors are Drs. Fred. A. Levy, of New Jersey, R. Finley Hunt, of Washington, and W. W. Walker.

---

OXYCYANIDE OF MERCURY, compared with the corrosive chloride.

I. Its solution has a slightly alkaline reaction, and precipitates albumen only slightly.

II. It is less irritant than solutions of sublimate.

III. There is less absorption by tissues than in case of sublimate.

IV. Solution 1-1500 does not attack, except slightly, the materials used in surgical instruments.

V. Vested by its power of keeping soup, the antiseptic power showed itself six times greater than that of bichloride.

VI. Vested by the power to destroy the micrococcus pyogenes aureus, the advantage was slightly in favor of bichloride, 1-1400 to 1-1300.

VII. Employed on suppurating surfaces, or to render a mucous surface antiseptic, it furnishes much better results, because of the tolerance by tissues and feeble absorption.

The cyanide of mercury has about the same properties, but the oxycyanide is more powerful against the micrococcus pyogenes aureus.

